# Supplementary material for: The intronic branch point sequence is under strong evolutionary constraint in the bovine and human genome
Source: Commun Biol. 2021 Oct 21;4:1206. doi: 10.1038/s42003-021-02725-7 (PMC8531310; doi:10.1038/s42003-021-02725-7)
Supplement: Supplementary file 3 — Description of Additional Supplementary Files [file 42003_2021_2725_MOESM3_ESM.pdf]

Description of Additional Supplementary Files

File name: Supplementary Data 1

Description: Description and variability of branch point sequences identified in the bovine genome using BPP. Source data to generate Figures 3 and 4.

File name: Supplementary Data 2

Description: European Nucleotide Archive accession numbers for raw DNA sequencing data used to establish the variant catalogue.

File name: Supplementary Data 3

Description: European Nucleotide Archive accession numbers for raw RNA sequencing data used to establish the splicing QTL mapping cohort.

File name: Supplementary Data 4

Description: Source data to generate Figure 1a.

File name: Supplementary Data 5

Description: Source data to generate Figure 1b.

File name: Supplementary Data 6

Description: Source data to generate Figure 1c.

File name: Supplementary Data 7

Description: Source data to generate Figure 2.

File name: Supplementary Data 8

Description: Source data to generate Figure 5.

File name: Supplementary Data 9

Description: Description and variability of branch point sequences identified in the human genome using BPP. Source data to generate Figure 6.

File name: Supplementary Data 10

Description: Source data to generate the Manhattan plots in Figure 7 and 8.

File name: Supplementary Data 11

Description: Source data to generate beeswarm plots in Figure 7 and 8.
