# Supplementary material for: The intronic branch point sequence is under strong evolutionary constraint in the bovine and human genome
Source: Commun Biol. 2021 Oct 21;4:1206. doi: 10.1038/s42003-021-02725-7 (PMC8531310; doi:10.1038/s42003-021-02725-7)
Supplement: Supplementary file 15 — Reporting Summary [file 42003_2021_2725_MOESM15_ESM.pdf]

nature research

Corresponding author(s): Naveen Kadri
Last updated by author(s): 2021/09/13

Reporting Summary

Nature Research wishes to improve the reproducibility of the work that we publish. This form provides structure for consistency and transparency in reporting. For further information on Nature Research policies, see our Editorial Policies and the Editorial Policy Checklist.

Statistics

For all statistical analyses, confirm that the following items are present in the figure legend, table legend, main text, or Methods section.

n/a	Confirmed	
☐	☒	The exact sample size (*n*) for each experimental group/condition, given as a discrete number and unit of measurement
☐	☒	A statement on whether measurements were taken from distinct samples or whether the same sample was measured repeatedly
☐	☒	The statistical test(s) used AND whether they are one- or two-sided<br>*Only common tests should be described solely by name; describe more complex techniques in the Methods section.*
☐	☒	A description of all covariates tested
☐	☒	A description of any assumptions or corrections, such as tests of normality and adjustment for multiple comparisons
☐	☒	A full description of the statistical parameters including central tendency (e.g. means) or other basic estimates (e.g. regression coefficient) AND variation (e.g. standard deviation) or associated estimates of uncertainty (e.g. confidence intervals)
☐	☒	For null hypothesis testing, the test statistic (e.g. *F*, *t*, *r*) with confidence intervals, effect sizes, degrees of freedom and *P* value noted<br>*Give P values as exact values whenever suitable.*
☒	☐	For Bayesian analysis, information on the choice of priors and Markov chain Monte Carlo settings
☒	☐	For hierarchical and complex designs, identification of the appropriate level for tests and full reporting of outcomes
☒	☐	Estimates of effect sizes (e.g. Cohen's *d*, Pearson's *r*), indicating how they were calculated

Our web collection on statistics for biologists contains articles on many of the points above.

Software and code

Policy information about availability of computer code

Data collection	NA
Data analysis	fastp, Burrows-Wheeler Aligner (BWA), Picard tools, Genome Analysis Toolkit (GATK), Beagle4.1, Genome-wide Complex Trait Analysis (GCTA), BPP, LaBranchoR, branchpointer, STAR, Leafcutter, RegTools, FastQTL, ggsashimi

For manuscripts utilizing custom algorithms or software that are central to the research but not yet described in published literature, software must be made available to editors and reviewers. We strongly encourage code deposition in a community repository (e.g. GitHub). See the Nature Research guidelines for submitting code & software for further information.

Data

Policy information about availability of data

All manuscripts must include a data availability statement. This statement should provide the following information, where applicable:

- Accession codes, unique identifiers, or web links for publicly available datasets
- A list of figures that have associated raw data
- A description of any restrictions on data availability

All raw sequencing data of cattle used in this study are available from the European Nucleotide Archive under accession numbers listed in supplementary Data 2. The human variant catalogue is available via gnomAD (https://gnomad.broadinstitute.org/blog/2019-10-gnomad-v3-0/).

Field-specific reporting

Please select the one below that is the best fit for your research. If you are not sure, read the appropriate sections before making your selection.

- [x] Life sciences
- [ ] Behavioural & social sciences
- [ ] Ecological, evolutionary & environmental sciences

For a reference copy of the document with all sections, see nature.com/documents/nr-reporting-summary-flat.pdf

Life sciences study design

All studies must disclose on these points even when the disclosure is negative.

Sample size	whole genome sequencing data of 266 cattle, sequenced at coverage 10-fold or higher were used to establish the variant catalogue. This catalogue with 29.4 million SNPs was sufficient to study the difference in evolutionary constraints on the routinely annotated genomic features.<br>sQTL mapping cohort consisting of 76 samples with an average number of 283 million RNA sequencing reads and 12.6-fold genome coverage permits detection of alternative splicing events even for lowly expressed genes.
Data exclusions	Samples sequenced at lower coverage (<10 fold) were excluded prior to the analysis.
Replication	Findings in the cattle data were replicated in human data and reported in the manuscript
Randomization	Not applicable, study did not include experimental groups
Blinding	Not applicable, study did not involve allocating samples to groups

Reporting for specific materials, systems and methods

We require information from authors about some types of materials, experimental systems and methods used in many studies. Here, indicate whether each material, system or method listed is relevant to your study. If you are not sure if a list item applies to your research, read the appropriate section before selecting a response.

Materials & experimental systems

n/a	Involved in the study	
x		Antibodies
x		Eukaryotic cell lines
x		Palaeontology and archaeology
	x	Animals and other organisms
	x	Human research participants
x		Clinical data
x		Dual use research of concern

Methods

n/a	Involved in the study	
x		ChIP-seq
x		Flow cytometry
x		MRI-based neuroimaging

Animals and other organisms

Policy information about studies involving animals; ARRIVE guidelines recommended for reporting animal research

Laboratory animals	NA
Wild animals	NA
Field-collected samples	No animals were sampled specifically for this study
Ethics oversight	No Ethics approval was required for this study

Note that full information on the approval of the study protocol must also be provided in the manuscript.

Human research participants

Policy information about studies involving human research participants

Population characteristics	NA
Recruitment	Publicly available genomic data (in anonymous form) from the GenomeAD database were used
Ethics oversight	NA

Note that full information on the approval of the study protocol must also be provided in the manuscript.
